# Differential Assimilation of Inorganic Carbon and Leucine by *Prochlorococcus* in the Oligotrophic North Pacific Subtropical Gyre

**DOI:** 10.3389/fmicb.2015.01401

**Published:** 2015-12-17

**Authors:** Karin M. Björkman, Matthew J. Church, Joseph K. Doggett, David M. Karl

**Affiliations:** Department of Oceanography and Daniel K. Inouye Center for Microbial Ecology: Research and Education, University of Hawaii, HonoluluHI, USA

**Keywords:** *Prochlorococcus*, photoheterotrophy, flow cytometric cell sorting, radioisotopes, North Pacific Subtropical Gyre, Station ALOHA

## Abstract

The light effect on photoheterotrophic processes in *Prochlorococcus*, and primary and bacterial productivity in the oligotrophic North Pacific Subtropical Gyre was investigated using ^14^C-bicarbonate and ^3^H-leucine. Light and dark incubation experiments were conducted *in situ* throughout the euphotic zone (0–175 m) on nine expeditions to Station ALOHA over a 3-year period. Photosynthetrons were also used to elucidate rate responses in leucine and inorganic carbon assimilation as a function of light intensity. Taxonomic group and cell-specific rates were assessed using flow cytometric sorting. The light:dark assimilation rate ratios of leucine in the top 150 m were ∼7:1 for *Prochlorococcus*, whereas the light:dark ratios for the non-pigmented bacteria (NPB) were not significant different from 1:1. *Prochlorococcus* assimilated leucine in the dark at per cell rates similar to the NPB, with a contribution to the total community bacterial production, integrated over the euphotic zone, of approximately 20% in the dark and 60% in the light. Depth-resolved primary productivity and leucine incorporation showed that the ratio of *Prochlorococcus* leucine:primary production peaked at 100 m then declined steeply below the deep chlorophyll maximum (DCM). The photosynthetron experiments revealed that, for *Prochlorococcus* at the DCM, the saturating irradiance (*E*_k_) for leucine incorporation was reached at approximately half the light intensity required for light saturation of ^14^C-bicarbonate assimilation. Additionally, high and low red fluorescing *Prochlorococcus* populations (HRF and LRF), co-occurring at the DCM, had similar *E*_k_ values for their respective substrates, however, maximum assimilation rates, for both leucine and inorganic carbon, were two times greater for HRF cells. Our results show that *Prochlorococcus* contributes significantly to bacterial production estimates using ^3^H-leucine, whether or not the incubations are conducted in the dark or light, and this should be considered when making assessments of bacterial production in marine environments where *Prochlorococcus* is present. Furthermore, *Prochlorococcus* primary productivity showed rate to light-flux patterns that were different from its light enhanced leucine incorporation. This decoupling from autotrophic growth may indicate a separate light stimulated mechanism for leucine acquisition.

## Introduction

*Prochlorococcus* is a numerically abundant cyanobacterium widely distributed throughout oligotrophic, tropical and subtropical marine ecosystems (Chisholm et al., 1992; Partensky et al., 1999; Partensky and Garczarek, 2010). Although its abundance and potential significance vary among oceans, *Prochlorococcus* generally dominates the picophytoplanktonic community in both cell numbers and biomass in the upper water column of the North Pacific Subtropical Gyre (NPSG), and typically attains cell abundances two orders of magnitude greater than those of *Synechococcus*, the second most abundant picophytoplankter in this ecosystem (Campbell et al., 1997). *Prochlorococcus* is also a significant contributor to primary productivity within the oligotrophic oceans (Goericke and Welschmeyer, 1993; Li, 1994; Liu et al., 1997; Partensky et al., 1999; Hartmann et al., 2014). Consequently, advancing the knowledge of what limits or promotes *Prochlorococcus* productivity, as well as its interaction with its biogeochemical environment has been, and still is, of great importance for gaining a comprehensive understanding of ecosystem function and regulation within Earth’s largest biomes.

Over the past two decades our understanding of the metabolic capabilities and life strategies of marine microbes have both greatly expanded and changed. With an increasing amount of information accumulating through genomic studies, as well as transcriptomics and proteonomics, novel organisms, metabolic pathways, and biological functions have been unveiled. This includes the discovery of proteorhodopsin (Béjà et al., 2001) for example, now recognized to be far more commonplace, and more broadly distributed among bacteria and archaea than originally hypothesized (Kolber et al., 2000; Campbell et al., 2008; DeLong and Béjà, 2010). These discoveries require the re-evaluation of microbial food web structure and ecosystem function (Karl, 2007, 2014) and highlight that our knowledge of marine ecosystems remains incomplete.

During the past few years emerging evidence of photoheterotrophy in cyanobacteria, including *Prochlorococcus*, has been reported. Originally, this photoheterotrophy was inferred from the observations of light stimulation of leucine incorporation (Paerl, 1991; Church et al., 2004, 2006; Michelou et al., 2007). In more recent field studies, as well as culture work, cell sorting techniques have indeed shown that *Prochlorococcus* assimilates amino acids (Zubkov et al., 2003; Michelou et al., 2007; Mary et al., 2008), and a recent study showed that photoheterotrophy in *Prochlorococcus* appears to be ubiquitous throughout the world’s surface oceans (Evans et al., 2015). However, the mechanism for this light supported uptake has not been determined (Zubkov, 2009). Furthermore, the potential contribution to ‘heterotrophic bacterial production’ estimates when using leucine incorporation as a proxy may need to be re-evaluated. The potential for indirect stimulation of growth of non-pigmented, heterotrophic bacteria through the utilization of freshly produced dissolved organic carbon during the daylight period is also of interest, particularly as the methods typically employed in ‘heterotrophic bacterial production’ measurements are conducted in the dark. Clearly, the ecological importance of light enhanced uptake of inorganic and organic materials in the open oceans has many implications for the understanding of the energy flux within the microbial communities.

Here we present work conducted during nine separate research cruises, over a 3-year period in the vicinity of, or at Station ALOHA in the NPSG. We used *in situ* incubations at eight discrete depths to assess both the light and dark ^3^H-leucine incorporation by the whole water microbial community. Our aim was to assess the magnitude of *Prochlorococcus* contribution to ‘heterotrophic bacterial production’ estimates over the full euphotic zone and on multiple occasions in this ecosystem, which until now has very limited data compared to other oceans. We further aimed to investigate the potential mechanism for the light enhanced leucine incorporation in *Prochlorococcus*. We hypothesized that leucine, when added at saturating concentrations, should be incorporated in lockstep with *Prochlorococcus* primary production, defined here as inorganic carbon reduction, if the light enhanced leucine incorporation is the reflection of autotrophic growth by *Prochlorococcus.*

## Materials and Methods

### Sample Location

Seawater sampling for depth profiles was conducted on several cruises in the NPSG from August 2007 through July 2010. The majority of these experiments was carried out at Station ALOHA (22.75°N, 158.00°W) on Hawaii Ocean Time-series (HOT) cruises, with additional experiments carried out on other research cruises within the NPSG, for a total of nine vertical profile experiments (**Table 1**). Seawater samples were collected using polyvinyl chloride (PVC) Niskin-type bottles mounted on a 24-place rosette frame and equipped with conductivity, temperature, and depth (CTD) sensors. On two occasions (November 2009, May 2013) water was collected for photosynthetron incubation experiments. Additional hydrographical and chemical data obtained from the CTD and discrete bottle samples, other instrumentation, or analysis, e.g., mixed layer depth (MLD), sea-surface temperature (SST), daily surface photosynthetically available radiation (PAR), chlorophyll *a* (chl *a*), nitrate concentrations, and whole water ^14^C-based primary production were obtained from the HOT program (HOT-DOGS; http://hahana.soest.hawaii.edu/hot/hot-dogs/interface.html).

**Table 1:** Cruise identification number, location (latitude and longitude), ambient sea surface temperature (SST), mixed layer depth (MLD), sea-surface photosynthetically available radiation (PAR), depth integrated (0–175 m) nitrate+nitrite (N+N), chlorophyll *a* (Chl *a*) concentration and primary production (PP) by ^14^C-bicarbonate incorporation.

Cruise	Date	Latitude/Longitude	SST (°C)	MLD (m)	PAR (mol quanta m^-2^ d^-1^)	N+N (mmol m^-2^)	Chl *a* (mg m^-2^)	PP (mmol C m^-2^ d^-1^)
KM0715#	16 August 2007	23°13′N, 159°08′W	26.6	50	38.7	42.0	40.0	65.0 ± 2.3
HOT 205	10 October 2008	22°45′N, 158°00′W	26.2	61 ± 7	39.2	50.6	33.8	42.7 ± 1.6
HOT 206#	30 November 2008	22°45′N, 158°00′W	25.0	78 ± 13	25.0	73.6	40.2	47.7 ± 1.7
HOT 209	17 February 2009	22°45′N, 158°00′W	22.6	100 ± 20	35.4	65.1	17.5	43.9 ± 1.2
HOT 210	28 April 2009	22°45′N, 158°00′W	22.7	55 ± 12	33.8	20.0	19.0	44.5 ± 1.1
HOT 213	24 July 2009	22°45′N, 158°00′W	24.2	49 ± 9	32.2	29.3	17.6	36.5 ± 0.7
KM1010-2	22 June 2010	19°55′N, 159°24′W	25.8	35	48.3	No data	28.8	39.8 ± 4.3
KM1010-3	23 June 2010	20°10′N, 158°46′W	25.8	35	43.4	No data	19.3	42.8 ± 2.2
KM1016#	27 August 2010	25°31′N 160°35′W	26.2	35	No data	No data	33.0	58.9 ± 3.3
HOT 216^∗^	3 November 2009	22°45′N, 158°00′W	25.6	55 ± 8	16.6	41.0	20.3	23.4 ± 0.7
KM1309^∗^	29 May 2013	22°45′N, 158°00′W	25.4	30	No data	21.2	22.2	No data


*SST in °C.*

* MLD determined by potential density offset of 0.125 from surface.*

*^∗^Photosynthetron incubation experiments.*

* #“bloom” conditions.*

### Depth Profile Incubation Experiments

Seawater was collected from 8 depths (5, 25, 45, 75, 100, 125, 150, 175 m), spanning the euphotic zone down to ≤0.2% of the surface PAR (Letelier et al., 2004). For each depth two 40 ml samples were placed into acid cleaned, DI and sample rinsed, polycarbonate tubes and inoculated with ^3^H-leucine (specific activity 3.7 or 4.2 TBq mmol^-1^; cat. #20032, MPBiomedicals) to effect a 20 nmol l^-1^ leucine addition. This concentration of leucine has been empirically determined to saturate ^3^H-leucine incorporation rates at Station ALOHA and has been used in previous studies to measure whole water community rates of leucine incorporation in this environment (Church et al., 2004). The incubations were conducted both in the light and dark, with one 40 ml sample for each depth placed inside a dark bag. Both the light and dark incubation bottles were mounted onto a free-floating, *in situ* array. In February 2009, triplicate 40 ml samples were placed at 45 and 125 m, respectively, to assess incubation variability. The light and dark bottles were placed at their respective depths of collection on the free-floating array to incubate at their natural temperature and light level. The array was deployed at dawn and recovered at dusk so that samples incubated over a full photoperiod. All ^3^H-leucine incorporation experiments were carried out in conjunction with the routine HOT primary production incubations by the ^14^C-bicarbonate method (standard HOT program protocol: ^14^C-bicarbonate; cat#17441H, MPBiomedicals, final activity approximately 3.7 MBq l^-1^, (Letelier et al., 1996)). In addition, ^14^C-bicarbonate incorporation by *Prochlorococcus* was conducted on two occasions (November 2008, February 2009) sampling the same eight depths as for the ^3^H-leucine experiments. These incubations were also carried out in 40 ml polycarbonate tubes spiked with a higher final activity of ^14^C-bicarbonate (137 MBq l^-1^) than the routine ^14^C-PP samples to be able to detect ^14^C-incorporation by *Prochlorococcus*. These samples were incubated in the light on the same *in situ* free-floating array as described above.

### Photosynthetron Experiments

Photosynthetrons (Lewis and Smith, 1983) were used to investigate the response of populations from a given depth to a range of light intensities using both ^14^C-bicarbonate and ^3^H-leucine. These experiments were conducted during cruises to Station ALOHA (November 2009, May 2013) using seawater samples collected within the mixed layer (25 m) and at the deep chlorophyll maximum (DCM; 125 m). The light intensity in the 24-well photosynthetron ranged from a few to ∼2000 μmol quanta m^-2^ s^-1^, the latter equivalent to the maximum full-sunlight at the surface of the ocean at local noon in summer. The range in light intensities bracketed the light flux the seawater samples would have experienced at their collection depth around noon (25 m November ∼250–300 μmol quanta m^-2^ s^-1^; 125 m, May and November, 5–10 μmol quanta m^-2^ s^-1^). Illumination was provided by dual 120V, 250W tungsten-halogen bulbs with dichroic reflectors (ENH-type: EIKO, Japan) providing a continuous light spectrum and a color temperature of 3250K. The light intensity was attenuated using neutral density filters to achieve the desired range of light levels. The light intensity in each well was measured prior to each experiment using a Biospherical QSL-100 PAR sensor. The photosynthetron incubation chambers were cooled by a circulating waterbath maintained at the *in situ* temperatures at the ML or DCM, respectively, throughout the incubation period. Clean, glass scintillation vials were used for the incubations, each vial containing 15 ml seawater subsamples labeled with either ^3^H-leucine or ^14^C-bicarbonate. Twelve vials for each tracer were placed into the same 24-well photosynthetron and irradiated for 2 h. Additional dark samples were incubated at the same temperatures and duration as the samples in the photosynthetron. After the end of the incubation period the samples were processed as described below.

### Sample Processing

After recovery of the *in situ* array, or at the termination of the photosynthetron incubations, duplicate 2–4 ml aliquots were subsampled from each incubation vessel, preserved with paraformaldehyde (PFA; final concentration 0.24%), flash frozen in liquid nitrogen and stored at -80°C until analyzed for cell specific assimilation of leucine or inorganic carbon. The ^3^H-leucine incubations were also sampled for the total microbial community leucine incorporation (total-Leu) following the protocol in Kirchman (2001). The ^14^C-bicarbonate incubations were sampled for whole water primary production by filtering 10 ml through a GFF filter (Whatman, nominal pore size 0.7 μm). The filters were acidified (1 ml, 2N HCl) and allowed to vent for 24 h prior to adding the scintillation cocktail (Ultima Gold LLT, Perkin-Elmer). The radioactivity was determined on a Perkin-Elmer Tricarb scintillation counter using existing instrument quench curves and transformed Spectral Index of the External standard (t-SIE) to obtain sample dpm.

### Cell Counting and Sorting

Cell enumeration and sorting were performed on an Influx Mariner flow cytometer. Fluorescent reference beads (1 μm diameter, Fluoresbrite, Polyscience) were added to each sample. The beads were also used to determine background radioactivity in sorted samples, as described below. The *Prochlorococcus* population was enumerated and sorted from unstained samples. *Prochlorococcus* cells were characterized on their forward scatter and red fluorescence signals, distinguished from *Synechococcus* by the phycoerythrin content (orange fluorescence) of the latter, and from pico-eukaryotic phytoplankton based on size and relative red fluorescence. For the enumeration and sorting of NPB, a second aliquot of each sample was stained with SYBR green I (Invitrogen, 0.01% v/v final concentration). Because the *Prochlorococcus* population cannot be uniquely distinguished in the SYBR stained samples in the upper water column, the NPB cell numbers or activities were determined as the difference between *Prochlorococcus* in unstained samples and the total SYBR positive cells. The Influx data acquisition used the Spigot software (Cytopeia), and cell numbers for *Prochlorococcus* and NPB were determined using the FlowJo software (Tree Star Inc.). Between 25,000 and 200,000 cells for *Prochlorococcus* or SYBR stained populations were sorted per sample depending on experiment and cell type, to achieve sufficient signal. Two sort streams were collected simultaneously, directly into separate scintillation vials (7 ml Snaptwist, Simport). The primary sort stream contained the microbial cells selected and the secondary the fluorescent reference beads. The vial containing the beads was used to account for radioactivity in the small volume of seawater associated with the sorted cells and was subtracted from the radioactivity obtained from the cells (DPM cell^-1^ – DPM bead^-1^). Samples containing ^14^C were acidified (0.5 ml 2N HCl), vented 24 h to remove unincorporated inorganic ^14^C, prior to adding scintillation cocktail. The activity per liter for *Prochlorococcus* or NPB was calculated as the mean per cell radioactivity multiplied by the total number of cells l^-1^, and converted to ^3^H-leu or ^14^C-bicarbonate incorporation rates as pmol leu l^-1^ h^-1^, or nmol C l^-1^ h^-1^, by their respective specific activities (Bq mol^-1^). The average per cell rate was also determined (amol leu or C cell^-1^ h^-1^).

From the photosynthetron experiments the parameters *P*_max_ and *E*_k_ were derived by fitting the data to the Platt et al. (1980) model, where *P*_max_ is the calculated maximum incorporation rate and *E*_k_ the light intensity at which light saturation of the incorporation is reached as *E*_k_ = *P*_max_/α, where α is the initial slope of the curve. When comparing ^3^H-leucine versus ^14^C-incorporation response as a function of light only the *E*_k_ was used.

## Results

### Mixed Layer and Deep Chlorophyll Maximum Depth, Temperature, Chlorophyll *a*, and Primary Production

The MLD varied from 30 to 100 m, with the majority of the *in situ* experiments conducted when the MLD was <55 m. The deepest MLDs (±SD, 4 days cruise average, *n* = 15) were recorded in February 2009 (100 ± 20 m) and November 2008 (78 ± 13 m). Sea surface temperature (SST) ranged from 22.6°C (February 2009) to 26.6°C (August 2010; mean 25.2 ± 1.4, *n* = 12; **Table 1**). Chl *a* concentrations (integrated 0–175 m) ranged from 17.5 mg chl *a* m^-2^ (February 2009) to 40.2 mg chl *a* m^-2^ (November 2008). The latter was the highest chl *a* inventory on record for Station ALOHA since the beginning of the HOT program in October 1988. Two additional experiments were conducted during elevated chl *a* conditions (**Table 1**; August 2007 and August 2010 at ≥40% above HOT long-term mean for August). In addition to elevated chl *a*, rates of primary production during these three cruises were significantly greater than the HOT program 25-years means for these months [**Table 1**: HOT long-term mean (mmol C m^-2^ d^-1^, ± SE): August 52.4 ± 2.3, *n* = 23; November 36.8 ± 2.4, *n* = 16]. These three experiments (August 2007, 2010, and November 2008) were considered to have been during ‘bloom’ conditions. During the cruises when photosynthetron experiments were carried out, the depth of the DCM varied 40–50 m (November 2009, 90–130 m; May 2013, 110–160 m). However, for both November and May samplings for the photosynthetron incubations, the DCM was located at 125 m.

### Cell Numbers and Bacterial Production Dynamics

**FIGURE 1:**
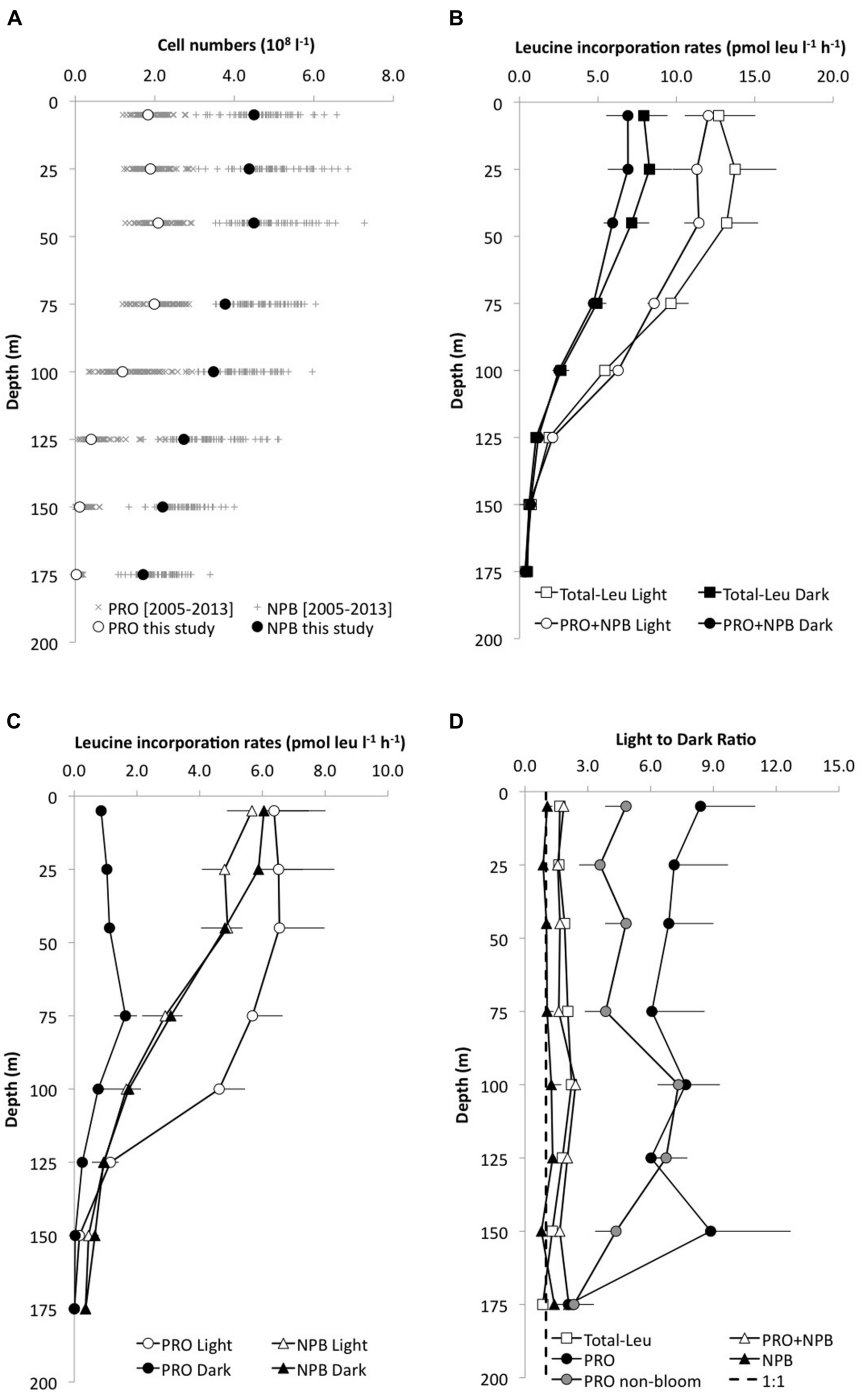
**Depth profiles of **(A)** the mean (±SD; *n* = 9) cell abundance of *Prochlorococcus* (PRO, open circles) and non-pigmented bacteria (NPB, filled circles) in the upper 175 m at Station ALOHA (this study), and the HOT long-term variability (2005–2013).** **(B)** leucine incorporation (pmol leu l^-1^ h^-1^) in the light (open circles) and dark (filled circles) of the total microbial community leucine incorporation (total-Leu, squares) or sorted PRO+NPB cells (circles); **(C)** leucine incorporation by PRO (circles) and NPB (triangles) in the light and dark and **(D)** the light:dark (L:D) incorporation ratios for the total-Leu (open square), PRO (filled circles), NPB (filled triangle), and PRO+NPB (open triangle), respectively. The dashed line represents the L:D 1:1 ratio. PRO L:D is depicted as the mean of all cruises (*n* = 8), and as “non-bloom” (gray circles, *n* = 5).

The depth distribution of *Prochlorococcus* and NPB showed cell abundances of approximately 1.8 × 10^8^ cells l^-1^ and 4 × 10^8^ cells l^-1^, respectively, in the upper 100 m of the water column. *Prochlorococcus* cell numbers declined rapidly to typically <5% of the near-surface abundances at 175 m and on average 12.3 ± 5.4% between 100 and 175 m. NPB populations also declined with increasing depth, but with much smaller changes, maintaining one third of its upper water column inventory between 100 and 175 m (**Table 2**; **Figure 1A**). This resulted in a variable NPB:*Prochlorococcus* ratio that increased from approximately 2.5 in the upper 100 m, to 6 at 125 m, to >50 at 175 m. This distribution is within the HOT longer term varibility (2005–2013; **Figure 1A**). Cell abundances integrated over the depth of the euphotic zone (0–175 m) varied among experiments for both *Prochlorococcus* and NPB and ranged from 13.4 to 25.1 ×10^12^ cells m^-2^ for *Prochlorococcus* (mean 20.8 ×10^12^± 3.5 ×10^12^ cells m^-2^, *n* = 9), and 48.2 to 74.2 ×10^12^ cells m^-2^ for NPB (mean 61.4 ×10^12^± 10.5 ×10^12^ cells m^-2^; **Table 2**). The lowest *Prochlorococcus* inventory (13.4 ×10^12^ m^-2^), observed in February 2009, coincided with the deepest mixing period. This is also consistent with the HOT program long-term record where February, on average, has the lowest *Prochlorococcus* cell abundances and deepest mixing at Station ALOHA.

**Table 2:** Cell numbers of *Prochlorococcus* (PRO) and non-pigmented bacteria (NPB) and relative distribution (%) of cell abundances and contribution to the total microbial community leucine incorporation (total-Leu) between 100 and 175 m.

Date	Cell numbers (×10^12^ m^-^^2^)		% of population (100–175 m)		% of total-Leu (100–175 m)	
			
	PRO	NBP	PRO	NPB	Light	Dark
August 2007	25.1	73.6	21.3	32.3	14.9	15.5
October 2008	23.5	74.2	8.4	28.4	9.7	8.7
November 2008	22.1	66.9	14.9	31.1	11.2	8.7
February 2009	13.4	53.7	13.4	33.9	15.0	15.5
April 2009	23.0	68.3	11.5	35.4	18.8	18.3
July 2009	21.1	66.0	5.9	24.7	6.0	5.6
June 2010	19.5	48.8	14.1	30.0	9.0	15.2
June 2010	18.2	48.2	8.9	28.1	3.7	4.1
August 2010	21.6	52.8	12.7	15.7	4.0	4.8


*Cell numbers are integrated between 0–175 m.*

Over the 3-years study period, total-Leu incorporation varied by a factor of five in the top 45 m, (range Light 4.2–20.6 pmol leu l^-1^ h^-1^; Dark 2.9–16.6 pmol leu l^-1^ h^-1^), averaging 11.9 ± 5.4 and 7.1 ± 3.4 pmol leu l^-1^ h^-1^, respectively, in the light and dark incubations (*n* = 24; **Figure 1B**). The total-Leu in the light was consistently and significantly higher (*p* < 0.0001, paired *t*-test, *n* = 54) than in the dark by a factor of 1.6–2.2 in the upper 125 m, then declining to a light to dark ratio of approximately 1 at 175 m (**Figure 1D**). The total-Leu was greatest during periods that coincided with the elevated primary production (the ‘bloom’ events). Depth integrated total-Leu (0–175 m), ranged from 0.63 ± 0.01 to 2.14 ± 0.03 μmol leu m^-2^ h^-1^ in the light, and from 0.34 ± 0.02 to 1.03 ± 0.04 μmol leu m^-2^ h^-1^ in the dark, averaging 1.26 ± 0.51 and 0.71 ± 0.25 μmol leu m^-2^ h^-1^ (*n* = 9), in the light and dark, respectively (**Table 3**). The integrated total-Leu (0–175 m) light to dark ratio (L:D) ranged from 1.4 ± 0.1 to 2.2 ± 0.1 μmol leu m^-2^ h^-1^ with mean ratio of 1.8 ± 0.2 (*n* = 9).

**Table 3:** Total microbial community (total-Leu) and group specific (*Prochlorococcus* [PRO], and non-pigmented bacteria [NPB]) ^3^H-leucine incorporation in the light (L) and dark (D).

Date	Total-Leu (μmol Leu m^-^^2^ h^-^^1^)		Group specific (μmol Leu m^-^^2^ h^-^^1^)				Light:Dark ratio		
			
	Light	Dark	PRO L	PRO D	NPB L	NPB D	Total-Leu	PRO	NPB
August 2007	1.66 ± 0.04	0.98 ± 0.02	0.66	0.11	0.45	0.47	1.7 ± 0.1	6.3	0.94
October 2008	2.14 ± 0.03	1.03 ± 0.04	No data	No data	No data	No data	2.1 ± 0.1	No data	No data
November 2008	1.71 ± 0.04	0.79 ± 0.02	1.50	0.08	No data	No data	2.2 ± 0.1	17.6	No data
February 2009	0.73 ± 0.04	0.34 ± 0.02	0.65	0.09	No data	No data	2.1 ± 0.1	7.0	No data
April 2009	0.90 ± 0.04	0.63 ± 0.02	0.27	0.19	0.84	0.48	1.4 ± 0.1	1.4	1.75
July 2009	0.63 ± 0.01	0.35 ± 0.02	0.50	0.12	0.15	0.32	1.8 ± 0.0	4.1	0.46
June 2010	1.22 ± 0.04	0.75 ± 0.01	0.61	0.18	0.44	0.50	1.6 ± 0.1	3.3	0.88
June 2010	0.98 ± 0.01	0.58 ± 0.01	0.57	0.16	0.40	0.41	1.7 ± 0.0	3.5	0.97
August 2010	1.42 ± 0.03	0.92 ± 0.02	0.77	0.12	0.48	0.75	1.6 ± 0.0	6.5	0.64


*Integrated over 0–175 m of the water column.*

The assessment of natural, field variability conducted on triplicate samples incubated at 45 and 125 m (February 2009) showed less than ± 10% variability in the total-Leu at 45 m, and < 20% at 125 m (7.8 ± 0.6 and 1.6 ± 0.2 pmol leu l^-1^ h^-1^, respectively) among triplicate incubations. However, the leucine incorporation by *Prochlorococcus* varied <1% at 45 m, whereas the 125 m showed similar variability as the total-Leu samples (7.6 ± 0.04 and 1.1 ± 0.2 pmol leu l^-1^ h^-1^, respectively).

### Group Specific Leucine Incorporation

The average light and dark leucine incorporation by sorted cells (*Prochlorococcus* +NPB) were not significantly different (paired *t*-test, *n* = 8) from those obtained by the TCA precipitation method for total-Leu, and the rates in the dark were very similar between these two measurements throughout the euphotic zone (**Figure 1B**). Leucine incorporation by *Prochlorococcus* was markedly higher in the light ranging from 6.8 ± 1.6 to 4.2 ± 0.8 pmol leu l^-1^ h^-1^ (SE, *n* = 8) in the top 100 m and then declined rapidly with reduced cell numbers and with depth. Dark leucine incorporation by *Prochlorococcus* ranged from 0.8 ± 0.2 (SE, *n* = 8) to 1.7 ± 0.4 (SE, *n* = 8) pmol leu l^-1^ h^-1^ from the surface to 100 m and then declined at greater depths (**Figure 1C**). Depth integrated rates (0–175 m) varied over the 3-years period by a factor of 5.5 and 2.2 for *Prochlorococcus* in the light and dark, respectively (**Table 3**). Leucine incorporation by NPB in the upper 175 m was, on average, not significantly different between light and dark incubations, although at 5 and 25 m a slightly lower rate was observed in the light. The rates ranged from 1.7 ± 0.9 to 5.7 ± 0.8 pmol leu l^-1^ h^-1^ in the light and 1.7 ± 0.9 to 6.0 ± 1.4 pmol leu l^-1^ h^-1^ (SE, *n* = 6) in the dark in the upper 100 m and then the rates for NPB declined to 175 m, although to lesser extent than *Prochlorococcus* (**Figure 1C**). Depth integrated rates (0–175 m) ranged from 0.15 to 0.84 μmol leu m^-2^ h^-1^ in the light and 0.32 to 0.75 μmol leu m^-2^ h^-1^ in the dark (**Table 3**). The contribution by *Prochlorococcus* to the total-Leu incorporation, integrated over the euphotic zone, amounted to approximately 62 ± 22% (*n* = 8) in the light and 22 ± 9% (*n* = 8) in the dark.

**FIGURE 2:**
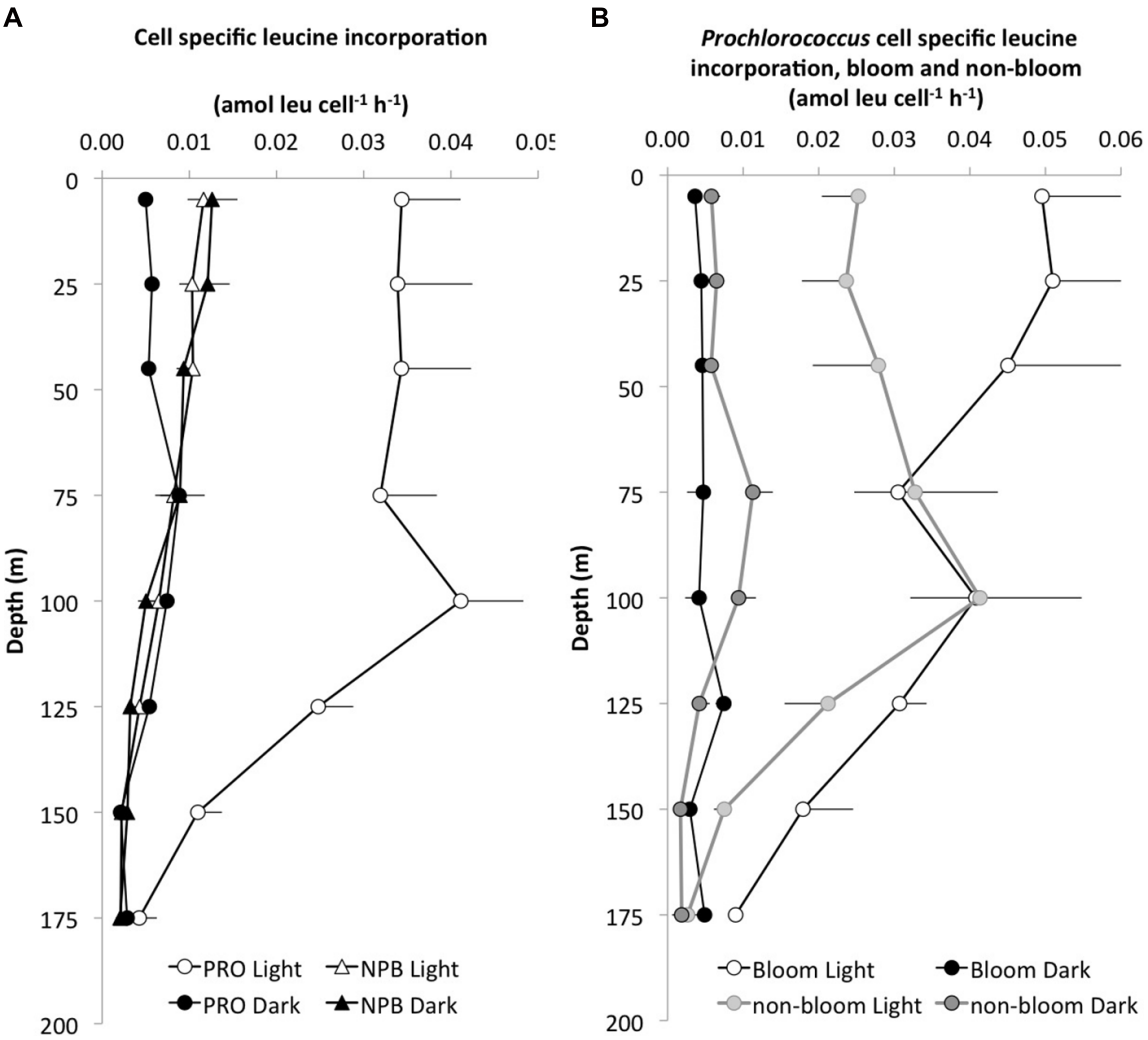
**Depth profiles of cell specific leucine incorporation rates (amol leu l^-1^ h^-1^).** **(A)** The average (±SE, *n* = 8) light (white symbols) versus dark (black symbols) incorporation by *Prochlorococcus* (PRO, circles) and NPB, triangles) and **(B)** cell specific incorporation in the light and dark by PRO during bloom (light gray, *n* = 3) and non-bloom (dark gray, *n* = 5) conditions.

The L:D incorporation ratios of *Prochlorococcus* were significantly higher than for total-Leu (paired *t*-test; *p* < 0.0001, *n* = 57) throughout the upper water column, and the ratio remained elevated down to 150 m (**Figure 1D**), whereas for NPB L:D incorporation ratios were not significantly different from 1:1. Both *Prochlorococcus* +NPB, and total-Leu L:D incorporation were significantly greater than 1:1 (paired *t*-test; *p* < 0.001, *n* = 32). The integrated L:D incorporation showed relatively little temporal variability for total-Leu, but L:D incorporation by *Prochlorococcus* varied by over an order of magnitude among cruises (**Table 3**). On a per cell basis, *Prochlorococcus* maintained relatively high rates of leucine incorporation in the light throughout the top 125 m of the water column ranging from 0.011 to 0.087 amol leu cell h^-1^ (mean 0.033 amol leu cell h^-1^, SE, 0.002, *n* = 48), before declining. NPB per cell rates were approximately one third of the *Prochlorococcus* per cell rates in the light (**Figure 2A**). In the top 45 m *Prochlorococcus* per cell rates in the dark were lower than NPB, however, below 75 m the rates were comparable to the NPB per cell rates (**Figure 2A**). *Prochlorococcus* leucine incorporation rates were enhanced by approximately twofold in the upper water column (0–45 m) during the bloom events compared to the non-bloom samplings. This effect was only seen in the light incubations (**Figure 2B**).

### Comparison of ^14^C-bicarbonate and ^3^H-leucine Incorporation

**FIGURE 3:**
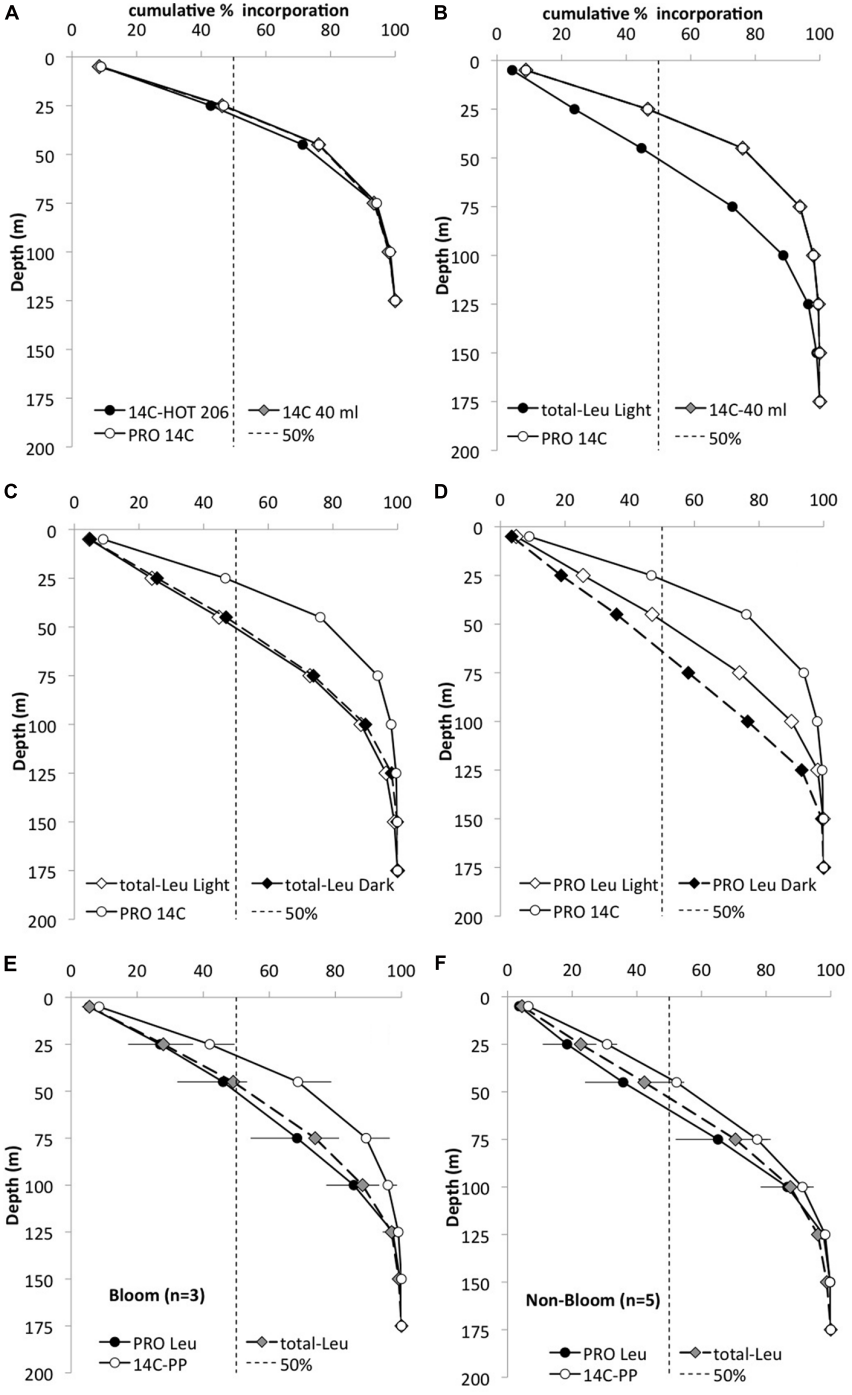
**Cumulative depth distribution (%) of the total integrated (0–175 m) ^14^C-bicarbonate (^14^C) and ^3^H-leucine (Leu) incorporation for November 2008.** Dashed line represents 50% of the total incorporation. **(A)** Whole water ^14^C primary production HOT core incubations (filled circles), whole water incubations for the cell sorting (gray diamonds), and ^14^C incorporation by *Prochlorococcus* (PRO; white circle) to 125 m. **(B)** Whole water ^14^C (gray diamond) and leucine incorporation (filled circle) and ^14^C-PRO (open circle) to 175 m; **(C)** whole water light (white diamonds) and dark (black diamonds) leucine incorporation and ^14^C-PRO (open circle); **(D)** PRO distribution for light (white diamonds) and dark (black diamonds) leucine and ^14^C (open circle) incorporation. **(E)** Mean PRO light leucine incorporation distribution (filled circle), total-Leu (gray diamonds) and ^14^C-primary production (open circle) incorporation during ‘bloom’ condition (*n* = 3) and **(F)** during ‘non-bloom’ condition (*n* = 5).

**FIGURE 4:**
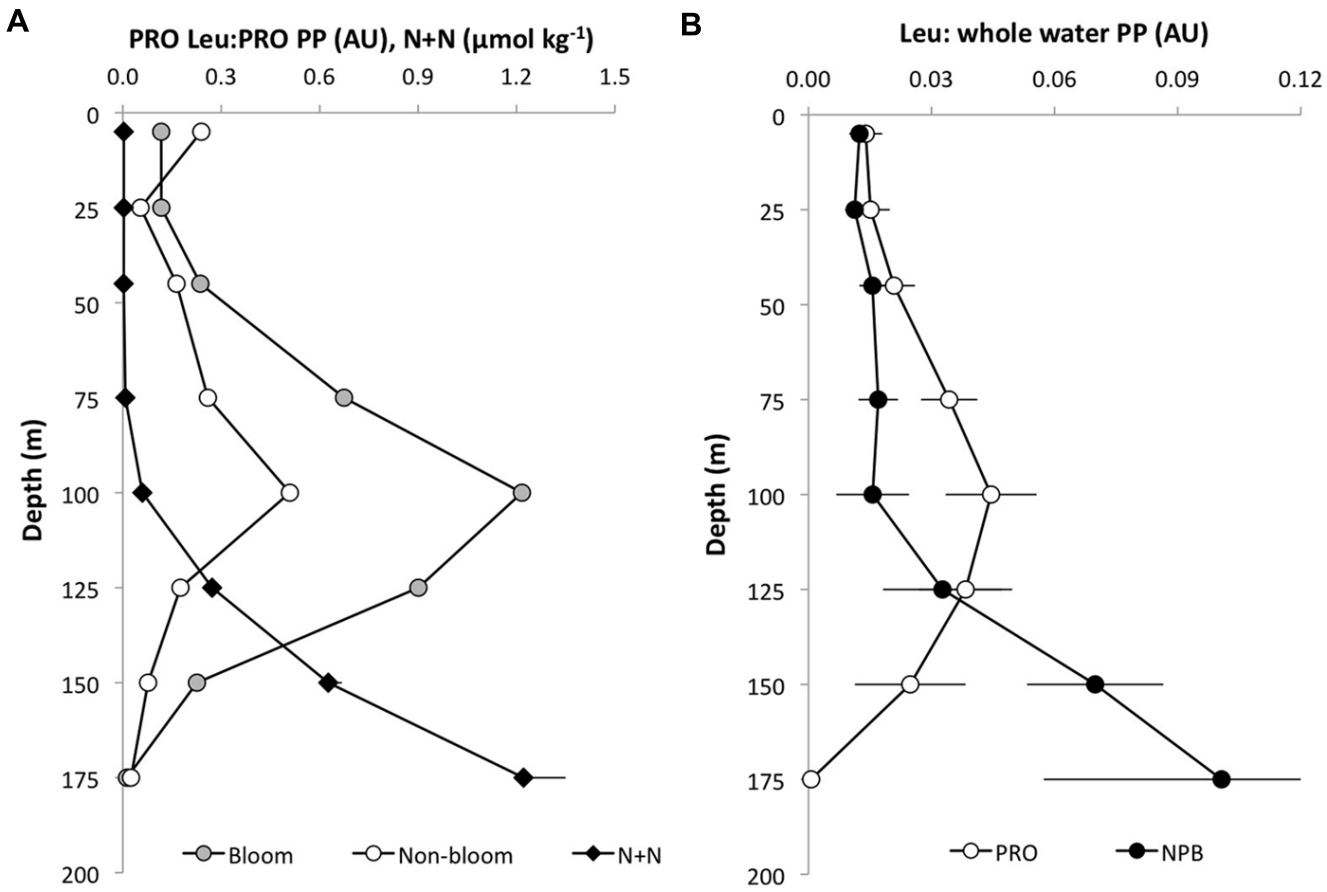
**Depth distribution of leucine incorporation to ^14^C-bicarbonate primary production (arbitrary units; AU) in **(A)***Prochlorococcus* (PRO Leu:PRO PP) November 2008 (bloom; gray circles) and July 2009 (non-bloom; open circles), and nitrite+nitrate concentrations (N+N; μmol kg^-1-^, black diamonds).** **(B)** PRO leu: community primary production (open circles) and NPB leu:community primary production (filled circles). Error bars are ± 1 SE of the mean (*n* = 8).

In November 2008 and February 2009, *Prochlorococcus* ^14^C-bicarbonate incorporation was also measured in conjunction with the ^3^H-leucine and the core HOT primary production experiments. In these experiments, ^14^C-*Prochlorococcus* appeared to contribute approximately 30–40% of the total ^14^C assimilated by the whole water community (GFF filters) in the upper 100 m, but the relative contribution by *Prochlorococcus* declined to ∼25% at 125 m and to <10% at greater depth. In order to compare the relative distribution of primary productivity to leucine incorporation the proportion of the total integrated primary production or leucine incorporation, was calculated as the cumulative fraction (%) at each depth throughout the euphotic zone (**Figure 3**). This cumulative distribution showed that ^14^C-assimilation by *Prochlorococcus*, whole water primary productions incubated in 40 ml vials, and the core HOT primary production all had very similar patterns (**Figure 3A**). By comparison, total-Leu showed a significantly different distribution from primary production (**Figure 3B**), whereas there was no significant difference between light and dark total-Leu (**Figure 3C**). However, *Prochlorococcus* leucine incorporation displayed marked differences between the light and dark depth distribution, and leucine also differed from the ^14^C-bicarbonate assimilation distribution patterns (**Figure 3D**). In these experiments, ^14^C-primary production always reached half of its total integrated production at a shallower depth than did leucine incorporation by *Prochlorococcus* or NPB, and during bloom events this depth separation was greater than during non-bloom events (**Figures 3E,F**). Furthermore, the rate ratio between ^3^H-leucine and ^14^C-bicarbonate incorporation showed that the relative contribution from leucine increased with depth with a maximum around 100 m, and then rapidly declined to the base of the euphotic zone. At the peak ratio *Prochlorococcus* leu: *Prochlorococcus* ^14^C-primary production was ∼ 5–10-fold higher compared to the surface depths (**Figure 4A**). The *Prochlorococcus* leu:community primary production ratio showed a similar distribution peaking around the DCM and a peak ratio about twice as high as that at surface depths, whereas NPB leu:community primary production had a relatively uniform distribution throughout the upper 100 m, and in contrast to the *Prochlorococcus* leu:community primary production, the NPB leu:community primary production increased at deeper depths (**Figure 4B**).

**FIGURE 5:**
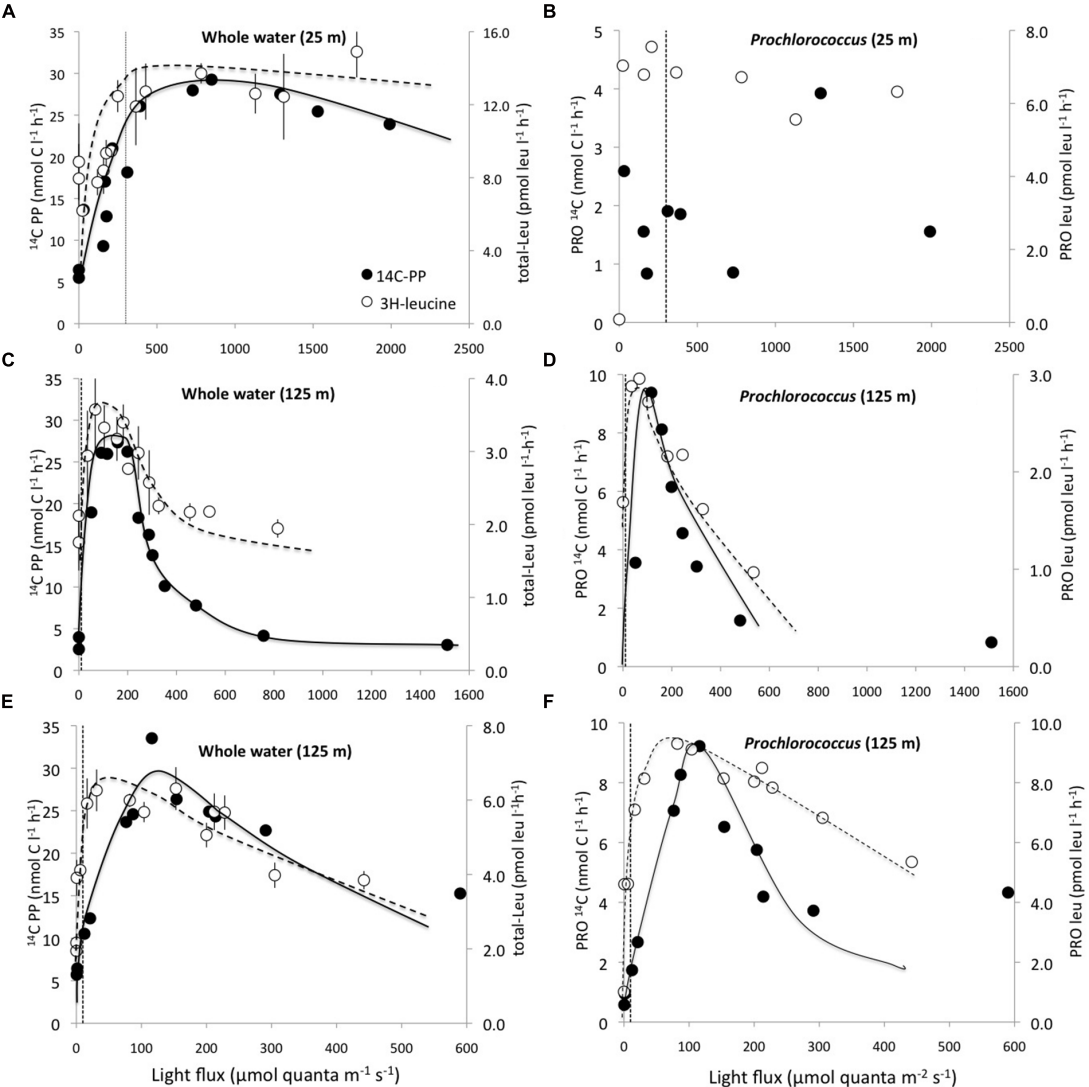
**Photosynthetron experiments depicting incorporation rates for ^14^C-bicarbonate (nmol C l^-1^ h^-1^; filled circles) and ^3^H-leucine (pmol leu l^-1^ h^-1^; open circles) as a function light intensity.** **(A)** 25 m whole water; **(B)** 25 m *Prochlorococcus*; **(C,E)** 125 m whole water; **(D,F)** 125 m *Prochlorococcus*. **(A–D)** November 2009 and **(E,F)** May 2013 experiments. Note that the scales on both x-, and y-axes differ among experiments. The dotted vertical lines represent the approximate light intensities at the depth of sample origin. Error bars for total community leucine incorporation (total-Leu) samples are ± 1 SD (*n* = 3).

**FIGURE 6:**
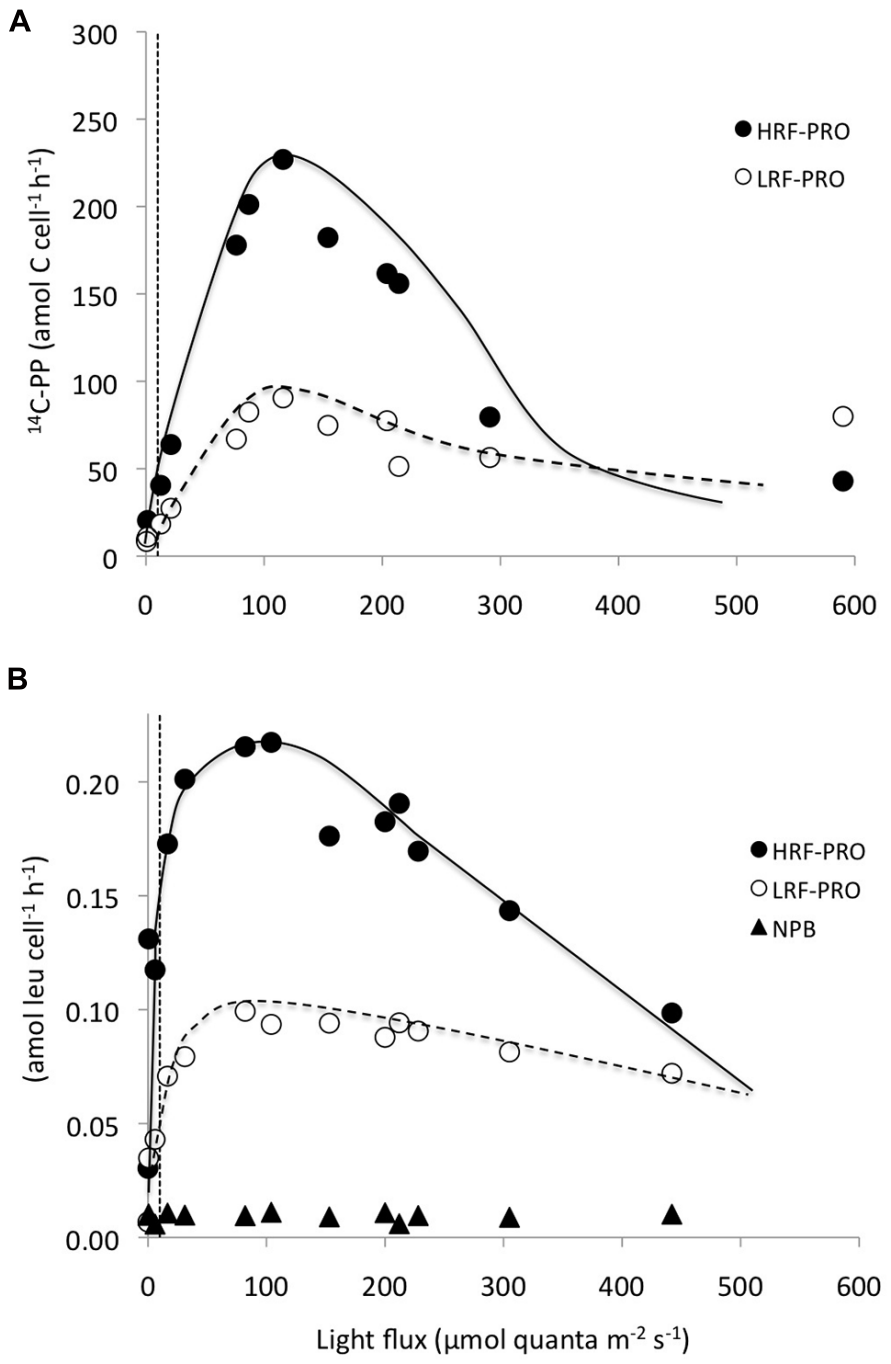
**Photosynthetron experiments showing per cell incorporation rates as a function light intensity by high red fluorescent (HRF: filled circles) and low red fluorescent (LRF: open circles) *Prochlorococcus* cells, co-occuring at the DCM.** **(A)** HRF and LRF ^14^C-bicarbonate assimilation rates (amol C cell^-1^ h^-1^) and **(B)** HRF, LRF, and NPB (filled triangles) ^3^H-leucine assimilation rates (amol leu cell l^-1^ h^-1^). The dotted vertical lines represent the approximate light intensities at the depth of sample origin.

In photosynthetron experiments conducted in November 2009 and May 2013, where ^3^H-leucine and ^14^C-bicarbonate assimilation rates were examined, both primary production and total-Leu responded to increasing light intensities. The mixed layer (25 m) sample revealed no apparent photoinhibition (**Figure 5A**), whereas the DCM (∼125 m) samples showed strong photoinhibition at a light flux above 150 μmol quanta m^-2^ s^-1^, and a very strong positive response with increased light only slightly above the light intensity experienced at the collection depth (**Figures 5C,E**). *Prochlorococcus* showed very similar dynamics as the whole water samples, with the exception of leucine, where *Prochlorococcus* was more strongly photoinhibited than the total-Leu (**Figures 5D,F**). There was no discernable light trend in the leucine incorporation by *Prochlorococcus* at 25 m, and ^14^C-assimilation was highly variable, due to lower than expected radioactivity of these sorted cells, and hence large potential errors associated with this data set (**Figure 5B**). Analysis of the photophysiological parameters showed that the leucine incorporation saturated at lower light intensities (lower *E*_k_ value) than ^14^C-primary production, for both the whole water community and *Prochlorococcus* samples, but the difference was larger for *Prochlorococcus* than for whole water samples (**Table 4**). *P*_max_ values for whole water primary production were remarkable similar for populations collected from 25 to 125 m (∼30 nmol C l^-1^ h^-1^), while ^3^H-leu *P*_max_ values were more variable (**Table 4**). In May 2013, two co-existing *Prochlorococcus* populations were distinguished at the DCM by their respective high and low relative red fluorescence (HRF and LRF, respectively). These two populations did not differ in their derived values of *E*_k_ but the light intensity for maximum leucine incorporation was approximately half of that required for maximum ^14^C-bicarbonate assimilation (∼40 versus ∼90 μmol quanta m^-2^ s^-1^, for leucine and ^14^C-bicarbonate, respectively). On a per cell basis, *P*_max_ rates of either leucine or ^14^C-bicarbonate incorporation, were approximately twice as high in HRF compared to LRF cells (**Figures 6A,B**). The photoinhibition was also markedly different with HRF population being more negatively affected at higher light flux than the LRF (**Figures 6A,B**). NPB per cell rates were relatively invariable at all light intensities tested and were markedly lower than for both HRF and LRF *Prochlorococcus* cells (**Figure 6B**).

**Table 4:** Photosynthetron experiments.

Date	Depth	*P*_max_		*E*_k_		α	
				
		^14^C	^3^H	^14^C	^3^H	^14^C	^3^H
	(m)	(nmol C l^-1^ h^-1^)	(pmol leu l^-1^ h^-1^)	(μmol quanta m^-2^ s^-1^)		^#^(xmol l^-1^ h^-1^)^∗^	(μmol quanta m^-2^ s^-1^)^-1^
**Whole water community**							
November 2009	25	29.1 ± 1.6	12.3 ± 0.8	483 ± 103	355 ± 42	0.06 ± 0.01	0.04 ± 0.00
	125	29.8 ± 1.6	3.6 ± 0.4	102 ± 14	59 ± 19	0.29 ± 0.03	0.06 ± 0.02
May 2013	125	28.2 ± 1.9	9.8 ± 1.4	70 ± 7	19 ± 6	0.40 ± 0.04	0.51 ± 0.15
***Prochlorococcus***							
November 2009	25	nd	7.1 ± 0.3	nd	nd	nd	nd
	125	9.6 ± 0.2	3.0 ± 0.1	118 ± 163	37 ± 1	0.08 ± 0.01	0.08 ± 0.00
May 2013	125	8.6 ± 0.1	9.9 ± 0.2	94 ± 10	26 ± 13	0.09 ± 0.00	0.37 ± 0.18
	HRF	5.4 ± 0.6	5.6 ± 0.2	88 ± 14	42 ± 11	0.06 ± 0.01	0.12 ± 0.03
	LRF	3.5 ± 0.4	4.2 ± 0.1	98 ± 11	30 ± 4	0.04 ± 0.00	0.14 ± 0.02


*Photophysiological parameters for maximum incorporation rate (*P*_*max*_), the light flux required to reach saturation (*E*_*k*_), and the initial slope (α) were derived from fitting the data to Platt et al. (1980) equation (*n* = 12). HRF, high red fluorescence, LRF, low red fluorescence *Prochlorococcus* cells. Nd, not determined.*

*^#^(xmol l^-1^ h^-1^) represents either (nmol C l^-1^ h^-1^) or (pmol leu l^-1^ h^-1^) for ^14^C and ^3^H, respectively.*

## Discussion

The capacity for photoheterotrophy in marine cyanobacteria, including *Prochlorococcus* and *Synechococcus*, has been known for many years (Paerl, 1991; Michelou et al., 2007; Mary et al., 2008; Zubkov, 2009). Field observations, using cell sorting techniques, have confirmed that *Prochlorococcus* can take up amino acids at near ambient concentrations (Zubkov et al., 2003; Evans et al., 2015) and that light enhances the assimilation (Michelou et al., 2007; Mary et al., 2008). Genomic and metagenomic studies also indicate that *Prochlorococcus* possesses amino acid transporters (Rocap et al., 2003; Partensky and Garczarek, 2010; Biller et al., 2015). However, most field studies have targeted population in the near-surface ocean, with full euphotic zone depth profiles being relatively rare. Furthermore, the great majority of these studies has been conducted in oceans other than the NPSG. Here, we present a multi-year study of light and dark leucine incorporation in the bulk water community and taxon specific groups of *Prochlorococcus* and NPB, as well as combined ^14^C-bicarbonate (primary productivity) and leucine incorporation experiments in order to investigate the light enhancement of leucine uptake and its relationship to ‘bacterial’ and primary productivity of *Prochlorococcus* in the oligotrophic NPSG ecosystem.

Pioneering work conducted by Church et al. (2004, 2006) at Station ALOHA in the NPSG, revealed strong light stimulation of ^3^H-leucine incorporation when comparing light and dark, *in situ* incubations of whole water samples. This light effect persisted throughout the water column, but the relative magnitude diminished with depth, with L:D ratios of approximately 1.5–2 from the surface waters down to 125 m, below which the ratio approached 1:1. The L:D ratios found within the upper 175 m were very similar to those observed here indicating that this is a persistent phenomenon within the NPSG ecosystem. Furthermore, we show here that this light enhancement in leucine incorporation is attributable to *Prochlorococcus*, which greatly increased its rate of leucine incorporation in the light. Although contributions from other picophytoplankton groups, such as *Synechococcus*, were not investigated, we found no significant difference between the absolute magnitude of the light enhancement (i.e., light–dark) observed in the whole water community to that measured for *Prochlorococcus*, suggesting that on average the community contribution from other picophytoplankton to the light-stimulated leucine incorporation was negligible at this study site. Our results may different from other oligotrophic marine environments, such as the subtropical North Atlantic Ocean, where *Prochlorococcus* typically constitutes a much smaller component of the picophytoplanktonic community than in the NPSG (Partensky et al., 1999).

Other investigators have reported high uptake rates of a variety of amino acids by *Prochlorococcus* (Zubkov et al., 2003), or by other cyanobacteria, as well as whole water communities, and both enhanced (Walsby and Juettner, 2006; Michelou et al., 2007; Mary et al., 2008; Evans et al., 2015) and suppressed (Morán et al., 2001) uptake of amino acids in the light have been observed. The contribution by *Prochlorococcus* in the light to the total-Leu was relatively high (∼60% of the total-Leu integrated over the euphotic zone) compared to the 13–24% reported from the North Atlantic (Michelou et al., 2007) and the ∼8% enhancement found in the Southern Atlantic Gyre, where tracer substrate additions were used (Evans et al., 2015). Notable, although *Prochlorococcus* showed much higher leucine incorporation rates in the light than in the dark, the dark rates were comparable, or higher, on a per cell basis than those of the NPB below 75 m, and in the surface waters reached 40–50% of the NPB per cell rates. Talarmin et al. (2011) also reported *Prochlorococcus* leucine per cell incorporation rates in the dark that were equal to, or exceeded, those of the NPB in the deeper portions of the euphotic zone in the Mediterranean Sea. Furthermore, *Prochlorococcus* near the DCM in the South Atlantic took up methionine at considerably higher rates then NPB (Zubkov et al., 2004). However, a recent study at Station ALOHA reported *Prochlorococcus* methionine uptake rates throughout the euphotic zone, that were lower, or on par with, the rates observed for the high and low nucleic acid containing NPB population, respectively (del Valle et al., 2015). Although the *Prochlorococcus* contribution to the dark total-Leu incorporation, integrated over the euphotic zone, was smaller (22 ± 9%), it is comparable to the estimates by Zubkov et al. (2003) from the Arabian Sea; but the dark contribution by *Prochlorococcus* reported from the North Atlantic were typically lower (5–14%; Michelou et al., 2007) than observed in the present study. Consequently, microbial leucine incorporation rates are not a unique measurement of ‘heterotrophic bacterial productivity,’ whether or not the incubations are conducted in the light or dark in these oligotrophic marine environments, and this needs to be taken into consideration when making assessments of carbon flux through the microbial food web.

The total-Leu incorporation rates declined nearly linearly below the mixed layer down to the DCM, with leucine incorporation in the light declining at twice the rate compared to changes in the dark. However, *Prochlorococcus* showed an almost uniform rate of leucine incorporation, on a per cell basis, from the surface to 75 m depth, an increase around the DCM, before rapidly diminishing to very low rates at the base of the euphotic zone. In comparison, NPB per cell leucine incorporation showed a more gradual decrease with increasing depth. This implies that leucine incorporation rates in *Prochlorococcus* saturate at relatively low light (LL) intensities. Church et al. (2006) came to a similar conclusion when investigating the light stimulation in the whole water community at Station ALOHA, but they were not at that time able to determine the mechanisms responsible for the effect. Zubkov et al. (2004) presented depth-resolved uptake of amino acids from the South Atlantic tropical gyre, and although their experimental design differed from ours in many respects (e.g., dark incubations, amino acid additions in the sub- to low nmol l^-1^ concentration range) they showed uptake rates of methionine in *Prochlorococcus* ranging fourfold between low and high red fluorescent (LRF and HRF, respectively) *Prochlorococcus* (0.6 ± 0.2 amol cell^-1^ d^-1^ in the upper 80 m, and 2.5 ± 1.6 amol cell^-1^ d^-1^ deeper within the euphotic zone). These per cell uptake rates were similar in magnitude to what we observed for leucine incorporation at saturating substrate concentrations in the light within the upper 100 m (mean 0.84 ± 0.08 amol cell d^-1^, *n* = 6). However, the increase in per cell rates observed at Station ALOHA between the near-surface populations and the maximum rates at 100 m were on average no more than 30% during *in situ* incubations. Nevertheless, the *Prochlorococcus* populations did shift from relatively small LRF cells to larger HRF cells around the DCM, presumably representing a shift in dominance between the high light (HL) and LL adapted ecotypes at these depths (Moore et al., 1998). Genomic and metagenomic studies at Station ALOHA have revealed a mixture of several members within the HL and LL clades, both presenting genotypic and phenotypic variability throughout the water column, and with varying contributions at different depths (Coleman and Chisholm, 2007; Malmstrom et al., 2010). Although we did not separate the LRF and HRF populations in the depth profile samples, the rate enhancement observed at the DCM could be the result of such co-existing HL and LL clades, where in fact the LL cells may have had substantially higher assimilation rates, as was indeed observed in the photosynthetron experiments (**Figures 6A,B**). The rates we measured for the HRF and LRF populations were more comparable to the rates found by Zubkov et al. (2004) mentioned above (this study photosynthetron sample at ∼6 μmol quanta m^-2^ s^-1^; LRF 1 amol leu cell d^-1^, HRF 3 amol leu cell d^-1^).

In the paired light incubations with ^14^C-bicarbonate and ^3^H-leucine, the ^14^C-primary production consistently attained half of its total euphotic zone production at shallower depths than leucine and the depth resolved relative contribution of leu:primary production showed that leucine incorporation by *Prochlorococcus* grew gradually more important with depth down to around the DCM, where this ratio peaked. This distribution may be the result of the interplay between light and nutrient availability, especially inorganic nitrogen, with light diminishing, and bioavailable nitrogen concentrations increasing. This is also the vertical stratum where the HRF and LRF *Prochlorococcus* populations change their respective dominance in the water column (Coleman and Chisholm, 2007), which likely also influences the leu:primary production ratio. The rates of primary production we derived for *Prochlorococcus* were comparable to those found by Li (1994) in the North Atlantic Ocean (e.g., at 60 m: 0.03–0.27 fg C cell^-1^ h^-1^; this study 75 m: 0.12–0.52 fg C cell^-1^ h^-1^), as well as with more recent work by Hartmann et al. (2014), reporting *Prochlorococcus* carbon fixation rates of ∼0.3–0.8 fg C cell^-1^ h^-1^ in the surface waters of the equatorial, north and south gyres of the Atlantic ocean. They concluded that surface *Prochlorococcus* contributes half of the primary production, slightly higher than the 30–40% we observed at Station ALOHA, and what Goericke and Welschmeyer (1993), reported from the Sargasso Sea (25% over all seasons, 30–40% during winter).

Photosynthetron experiments revealed a remarkably similar ^14^C-based *P*_max_ for surface and DCM populations. However, the *Prochlorococcus* per cell assimilation at the DCM was at least an order of magnitude higher than in the surface demonstrating the light limited conditions of cells at the base of the euphotic zone, as well as their ability to rapidly capitalize on increased light availability. These dynamics have previously been reported for *Prochlorococcus* by Moore and Chisholm (1999). They also showed that populations of *Prochlorococcus* isolated from the DCM maintained their photophysiological characteristics in culture and were comparable to wild populations. Their results in terms of *P*_max_ and *E*_k_ were similar to those reported here (*E*_k_ 20–90 μmol quanta m^-2^ s^-1^). The photosynthetron experiments showed low rates of leucine incorporation by the NPB component at 125 m (∼10 and 30% of the cell specific rates of the HRF and LRF populations, respectively), possibly reflecting their smaller size, and/or lower growth rates. Furthermore, inhibition at HL intensities was observed, similar to what typically has been demonstrated for primary production in LL adapted phytoplankton. In a multi-year study at Station ALOHA, Church et al. (2006) derived *E*_k_ values from the photo-stimulation of leucine incorporation that were also within the same range as we observed in our photosynthetron experiments. This supports the hypothesis that they presented at the time that *Prochlorococcus* is responsible for the majority of the increased leucine incorporation in the light.

A possible mechanism for light enhanced uptake of amino acids, and other compounds by *Prochlorococcus*, may be through the generation of ATP, via electron cycling in photosystem I (PSI). Paerl (1991) showed that amino acid uptake in the light was not affected by the treatment with photosystem II (PSII) inhibitors and concluded that the cyclic electron flow of PSI generating ATP could potentially be used to fuel cross-membrane transport systems. A recent study concluded that the RubisCO to PSII content in *Prochlorococcus* was low by comparison to its close relative *Synechococcus*, and that the rate-limiting step in *Prochlorococcus* photosynthesis likely was caused by the relatively low proportion of RubisCO (Zorz et al., 2015). This may create a bottle-neck in C-fixation and as a consequence much of the light energy captured could be funneled through PSI’s cyclic electron flow, generating ATP in the process. Having a relatively inefficient C-fixation process may therefore allow for excess light energy to be converted into ATP even at relatively LL intensities, which can be utilized to drive energy requiring cross-membrane transport. Such a mechanism would aid in nutrient acquisition as well as potentially supplementing organic compounds for the cell without restricting energy flow required for carbon fixation, or necessitating catabolic processes.

## Author Contributions

KB and KD performed the field work. KB wrote the manuscript. All authors contributed significantly in the preparation of the manuscript and in the interpretation of the data. All authors approve of the submission of this manuscript.

## Conflict of Interest Statement

The authors declare that the research was conducted in the absence of any commercial or financial relationships that could be construed as a potential conflict of interest.
